# Inhibition of monocarboxylate transporters (MCT) 1 and 4 reduces exercise capacity in mice

**DOI:** 10.14814/phy2.15457

**Published:** 2022-09-06

**Authors:** Yu Kitaoka, Kenya Takahashi, Hideo Hatta

**Affiliations:** ^1^ Department of Human Sciences Kanagawa University Kanagawa Japan; ^2^ Department of Sports Sciences The University of Tokyo Tokyo Japan

**Keywords:** exercise, lactate, MCT, skeletal muscle

## Abstract

The concept of lactate shuttle is widely accepted in exercise physiology. Lactate transport is mediated by monocarboxylate transporters (MCT), which enable cells to take up and release lactate. However, the role of lactate during exercise has not yet been fully elucidated. In this study, we investigated the effects of lactate transport inhibition on exercise capacity and metabolism in mice. Here, we demonstrated that MCT1 inhibition by α‐cyano‐4‐hydroxycinnamate administration (4‐CIN, 200 mg/g of body weight) reduced the treadmill running duration at 20 m/min. The administration of 4‐CIN increased the blood lactate concentration immediately after exercise. With matched exercise duration, the muscle lactate concentration was higher while muscle glycogen content was lower in 4‐CIN‐administered mice. Further, we showed that MCT4 inhibition by bindarit administration (50 mg/kg of body weight) reduced the treadmill running duration at 40 m/min. Bindarit administration increased the muscle lactate but did not alter the blood lactate and glucose concentrations, as well as muscle glycogen content, immediately after exercise. A negative correlation was observed between exercise duration at 40 m/min and muscle lactate concentration immediately after exercise. Our results suggest that lactate transport via MCT1 and MCT4 plays a pivotal role in sustaining exercise.

## Graphical abstract

In the current study, we examined whether selective inhibitors of MCT1 and MCT4 alter the exercise capacity and lactate metabolism. Our findings of reduced exercise duration after MCT inhibition indicate that lactate transport plays a pivotal role during exercise.

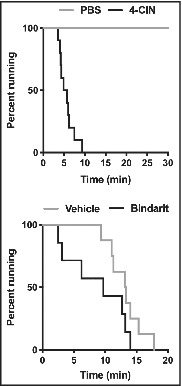

## 1 INTRODUCTION

At the onset of exercise, the demand for adenosine triphosphate (ATP) is increased many‐fold from rest in skeletal muscle (Hargreaves & Spriet, 2020). Since ATP availability is critical for muscle contractile activity and thus athletic performance, muscle glycogen is rapidly broken down, resulting in the production of lactate. Following all‐out maximal exercise, blood lactate concentration can elevate above 20 mM, which is markedly higher than that of glucose (Goodwin et al., 2007). Lactate was once thought to be a metabolic waste product, but it is now known that oxidation is the major fate of lactate, especially during exercise (Brooks, 2018). Importantly, skeletal muscle is not merely the site of lactate production, but has the ability to switch quickly from “producer” to “consumer” of lactate during exercise (van Hall, 2010). Moreover, increased blood lactate concentration with exogenous infusion was shown to increase lactate oxidation during exercise while it spared blood glucose, indicating that lactate is preferred over glucose as a fuel (Miller et al., 2002).

The release and uptake of lactate are mediated by monocarboxylate transporters (MCTs), which are widely expressed in various tissues including the liver, kidney, brain, and heart (Bonen et al., 2006). Lactate transport via MCTs is bi‐directional, depending on the concentration gradient of lactate and protons (Brown & Brooks, 1994; Juel, 1996). The most important isoforms in skeletal muscle are MCT1 and MCT4; MCT1 has high‐affinity for lactate and is mainly expressed in oxidative fibers, whereas MCT4 has low‐affinity for lactate and is present in glycolytic fibers (Bonen, 2001; Hashimoto et al., 2005). Previous studies have shown that exercise training increases the protein levels of MCT1 and MCT4 in the skeletal muscles of rodents (Coles et al., 2004), Thoroughbred horses (Kitaoka et al., 2011), and humans (Burgomaster et al., 2007), which leads to increased lactate shuttling. However, it is not clear whether the inhibition of these transporters alters lactate metabolism during exercise and thereby exercise capacity. In this study, we aimed to assess the contribution of MCT1 and MCT4 during exercise by using isoform‐specific inhibitors.

## 2 MATERIALS AND METHODS

### 2.1 Animals

Male ICR mice (8–10 weeks of age; CLEA Japan, Tokyo, Japan) were used in this study. Mice were housed on a 12:12‐h light–dark cycle (dark: 7:00 am to 7:00 pm) in an air‐conditioned room (22°C). All mice were provided standard chow (MF; Oriental Yeast, Tokyo, Japan) and water ad libitum during the experimental period. All the experiments were approved by the Animal Experimental Committee of the University of Tokyo (27–14).

### 2.2 Experimental design

#### 2.2.1 Experiment 1:4‐CIN Administration study

*Experiment 1–1*. Animals (*n* = 8) received 200 mg/g of body weight of α‐cyano‐4‐hydroxycinnamate (4‐CIN) via intraperitoneal injection. A solution of 4‐CIN was dissolved in 26% 1 M NaOH, as used in previous studies (Del Prete et al., 2004; Porporato et al., 2012; Sonveaux et al., 2008). Blood lactate concentration was measured over 180 min following 4‐CIN injection.

*Experiment 1–2*. Animals were assigned to two groups: phosphate‐buffered saline (PBS) or 4‐CIN administered (*n* = 6 each). Fifteen minutes after the intraperitoneal injection of PBS or 4‐CIN (200 mg/g body weight), animals received sodium lactate (1 mg/g body weight) via intraperitoneal injection, and the blood lactate concentration was measured over 180 min for the lactate tolerance test (LTT).

*Experiment 1–3*. Animals were assigned to the PBS or 4‐CIN administered exercise groups (*n* = 10 each). Fifteen minutes after the administration of PBS or 4‐CIN (200 mg/g body weight), the animals were subjected to treadmill running test at a speed of 20 m/min. Time to exhaustion was recorded when the mice were unable to maintain the running speed despite contacting the electrical grid for 5 consecutive seconds or 30 min. Blood lactate levels were measured at the end of the exercise.

*Experiemnt 1–4*. Animals were assigned to PBS or 4‐CIN administered exercise groups (*n* = 10 each), and paired randomly into different groups. Fifteen minutes after the administration of PBS or 4‐CIN (200 mg/g of body weight), the mice were subjected to treadmill running at 20 m/min. Since 4‐CIN‐administered mice ran less than PBS‐administered mice in Experiments 1–3, 4‐CIN‐administered mice were subjected to the running test first, followed by the PBS‐administered group, for matching the exercise duration in each pair. Blood lactate and glucose levels were measured at the end of the exercise.

#### 2.2.2 Experiment 2: Bindarit administration study

Animals received either bindarit (50 mg/kg body weight) or 0.5% methylcellulose vehicle via intraperitoneal injection (*n* = 8 each; Futagi et al., 2018; Zoja et al., 1998). Fifteen minutes after administration, the animals were subjected to treadmill running at a speed of 40 m/min until exhaustion. Time to exhaustion was recorded, and blood lactate and glucose levels were measured at the end of the exercise.

### 2.3 Tissue harvesting

Mice were euthanized by blood removal from the inferior vena cava under isoflurane inhalation immediately after exercise (Experiments 1–4 and 2). The plantaris and soleus muscles were quickly collected, snap‐frozen in liquid nitrogen, and stored at −80°C.

### 2.4 Blood and muscle analysis

The tail vein blood lactate and glucose levels were measured using Lactate Pro 2 (Arkray) and GLUCOCARD Plus Care (Arkray), respectively. Muscle lactate concentration was measured as described elsewhere (Takahashi et al., 2020). In brief, neutralized samples were mixed with assay solution (0.4 M hydrazine, 0.5 M glycine, 0.4 mM NAD+, and 1000 U l‐lactate dehydrogenase). After incubation for 30 min at room temperature, the absorbance at 340 nm was measured. Muscle glycogen content was measured as previously described (Takahashi et al., 2022). Briefly, the muscle samples were heated at 100°C in 30% KOH solution saturated with Na_2_SO_4_, mixed with 99.5% ethanol, and then centrifuged at 10,000 × g for 10 min at 4 °C. The pellets were hydrolyzed to glucose in 1 M HCl at 100 °C for 2 h and neutralized with 1 M NaOH. Glycogen content was determined using a glucose CII kit (Fujifilm Wako).

### 2.5 Statistical analysis

Values are reported as mean ± standard error of means (SEM). Comparisons between the two groups were analyzed using unpaired *t*‐tests. Correlations between two variables were examined using least‐squares linear regression followed by Pearson's correlation coefficient test. For the time course of blood lactate concentration, one‐way analysis of variance (ANOVA) was performed, followed by Dunnett's multiple comparisons test in Experiment 1–1, and two‐way ANOVA was performed followed by Bonferroni's multiple comparison test in Experiment 1–2. Running time in the treadmill test was analyzed using the log‐rank (Mantel–Cox) test. All statistical analyses were performed using GraphPad Prism 9 (GraphPad Software). Statistical significance was defined as *p* < 0.05.

## 3 RESULTS

### 3.1 Experiment 1: Effect of 4‐CIN administration

**FIGURE 1:**
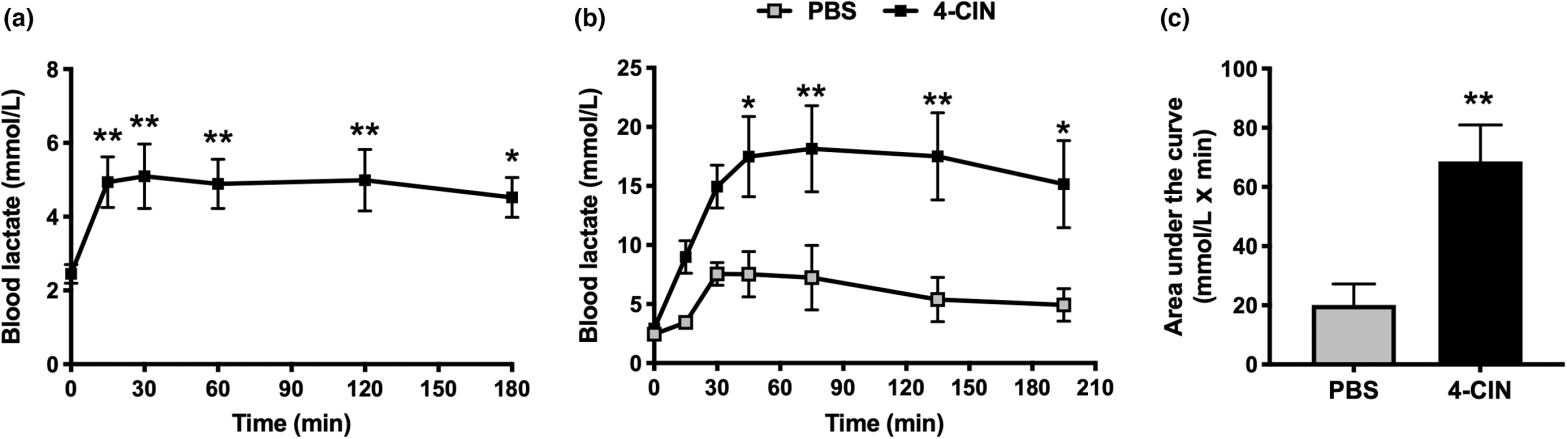
(a) Changes in blood lactate concentration following 4‐CIN (α‐cyano‐4‐hydroxycinnamate) injection assessed in a time‐dependent manner in Experiment 1‐1, (b) changes in blood lactate concentration, and (c) area under the curve in lactate tolerance test assessed in a time‐dependent manner in Experiment 1‐2. Mice received sodium lactate 15 min after injection of PBS or 4‐CIN. Data are presented as mean ± SEM. *N* = 6–8 in each group. **p < 0.01, *p < 0.05 vs PBS.

**FIGURE 2:**
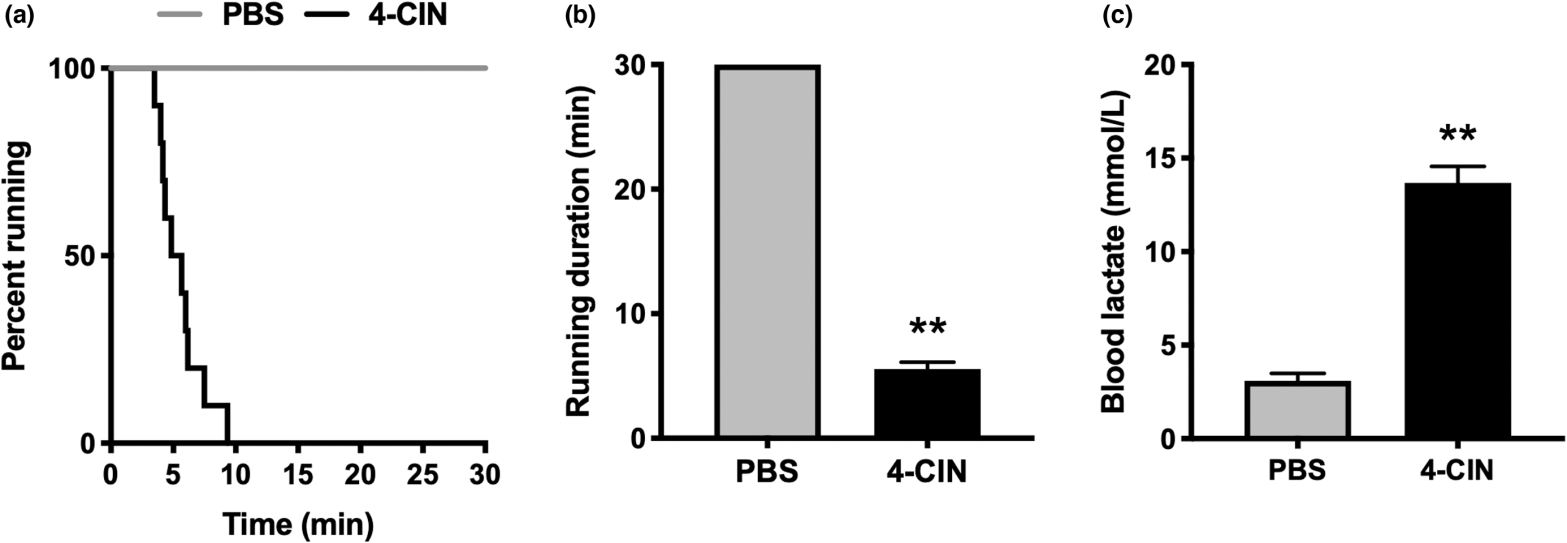
(a) Running survival curves and (b) average running duration assessed in treadmill running test at a speed of 20 m/min, and (c) blood lactate concentration measured after exercise in Experiments 1–3. Data are presented as mean ± SEM. *N* = 10 in each group. **p < 0.01 vs PBS.

**FIGURE 3:**
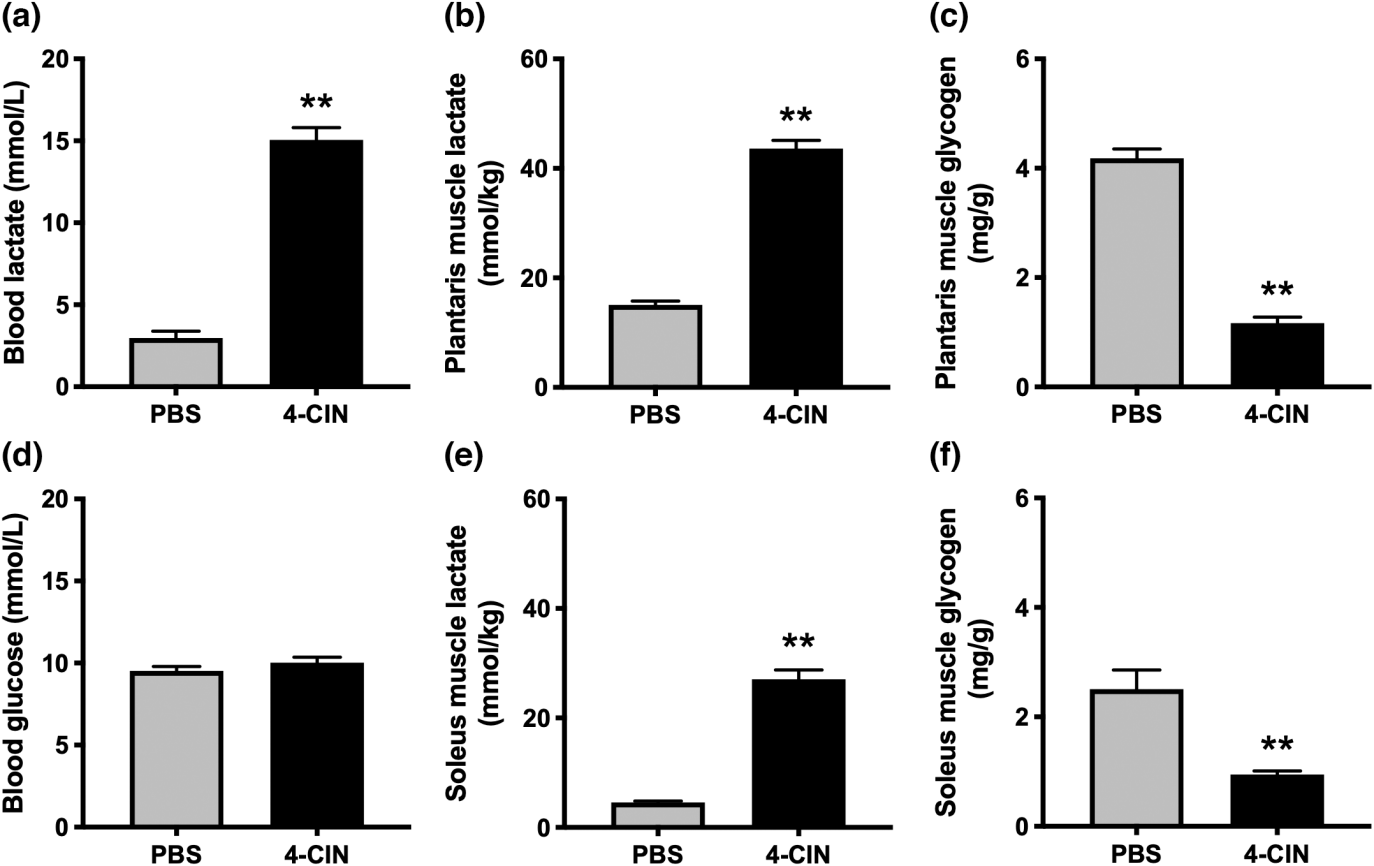
Blood concentrations of (a) lactate and (d) glucose, muscle lactate concentrations in (b) plantaris and (e) soleus, and muscle glycogen content in (c) plantaris and (f) soleus after exercise in Experiments 1–4. Data are presented as mean ± SEM. *N* = 10 in each group. **p < 0.01 vs PBS.

First, we assessed the time‐dependent changes in blood lactate concentration following 4‐CIN injection which were significantly increased 15 min after 4‐CIN injection and remained elevated over 180 min (Figure 1a). Next, we performed LTT with and without 4‐CIN administration. Blood lactate concentration was significantly higher in 4‐CIN‐administered mice from 30 to 180 min after lactate injection (Figure 1b), and the area under the curve was significantly higher in 4‐CIN‐administered mice (Figure 1c). In the treadmill running test at 20 m/min, there was a significant difference in the running survival curves (Figure 2a). Whereas PBS‐administered mice completed 30 min of running, 4‐CIN‐administered mice reached exhaustion before 30 min (Figure 2b). The blood lactate concentration after exercise was significantly higher in 4‐CIN‐administered mice than that after PBS administration (Figure 2c). The result of blood lactate was confirmed when the exercise duration was matched (Figure 3a), whereas there was no difference in blood glucose levels between the groups (Figure 3d). Following exercise, muscle lactate concentrations were significantly higher in 4‐CIN‐administered mice in the plantaris (Figure 3b) and soleus (Figure 3e), while muscle glycogen content was significantly lower in the plantaris (Figure 3c) and soleus (Figure 3f), compared to that in PBS‐administered mice.

### 3.2 Experiment 2: Effect of bindarit administration

**FIGURE 4:**
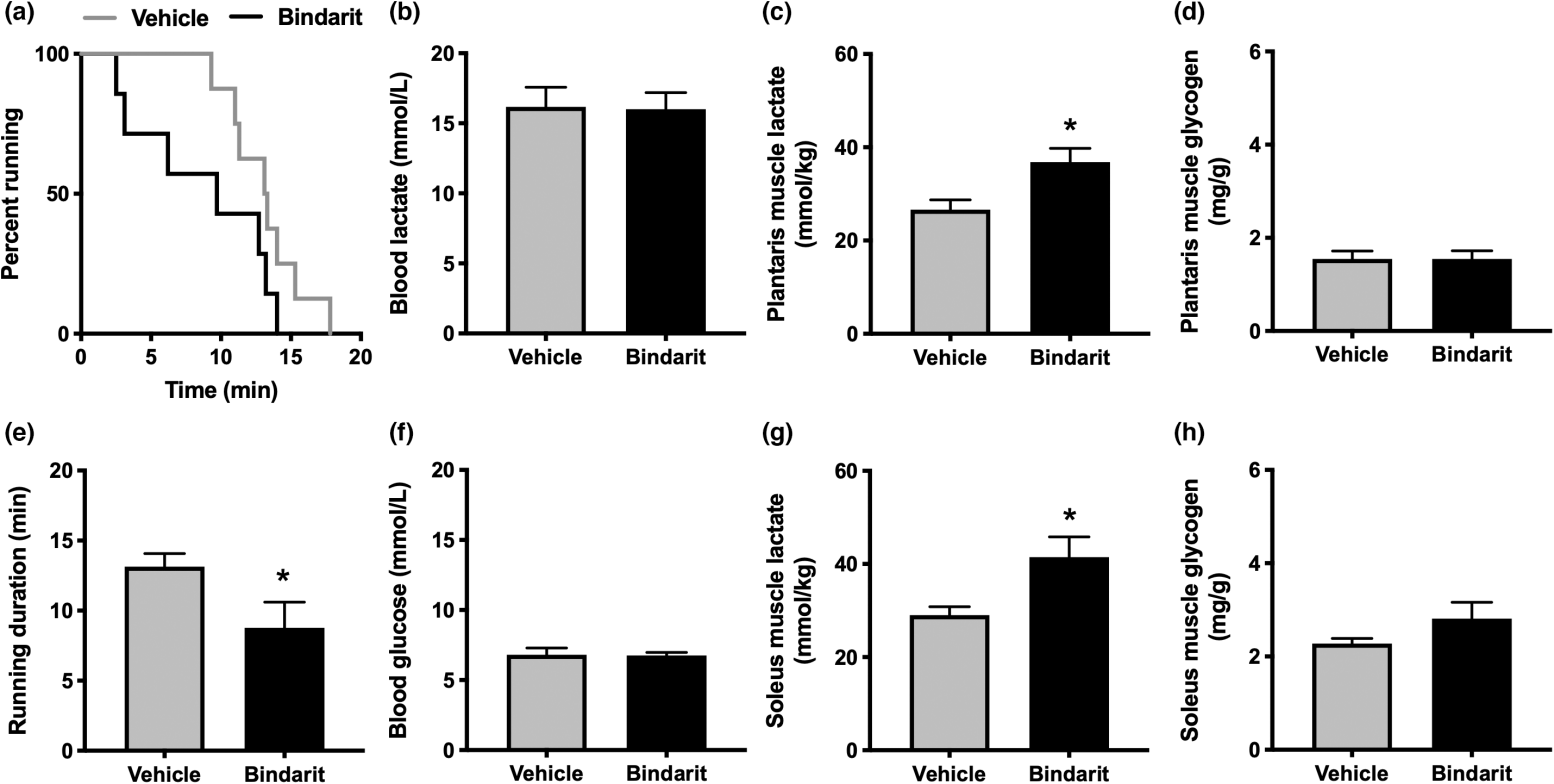
(a) Running survival curves and (e) average running duration assessed in treadmill running test at a speed of 40 m/min, blood concentrations of (b) lactate and (f) glucose, muscle lactate concentrations in (c) plantaris and (g) soleus, and muscle glycogen content in (d) plantaris and (h) soleus, after exercise in Experiment 2. Data are presented as mean ± SEM. *N* = 7–8 in each group. *p < 0.05 vs vehicle.

**FIGURE 5:**
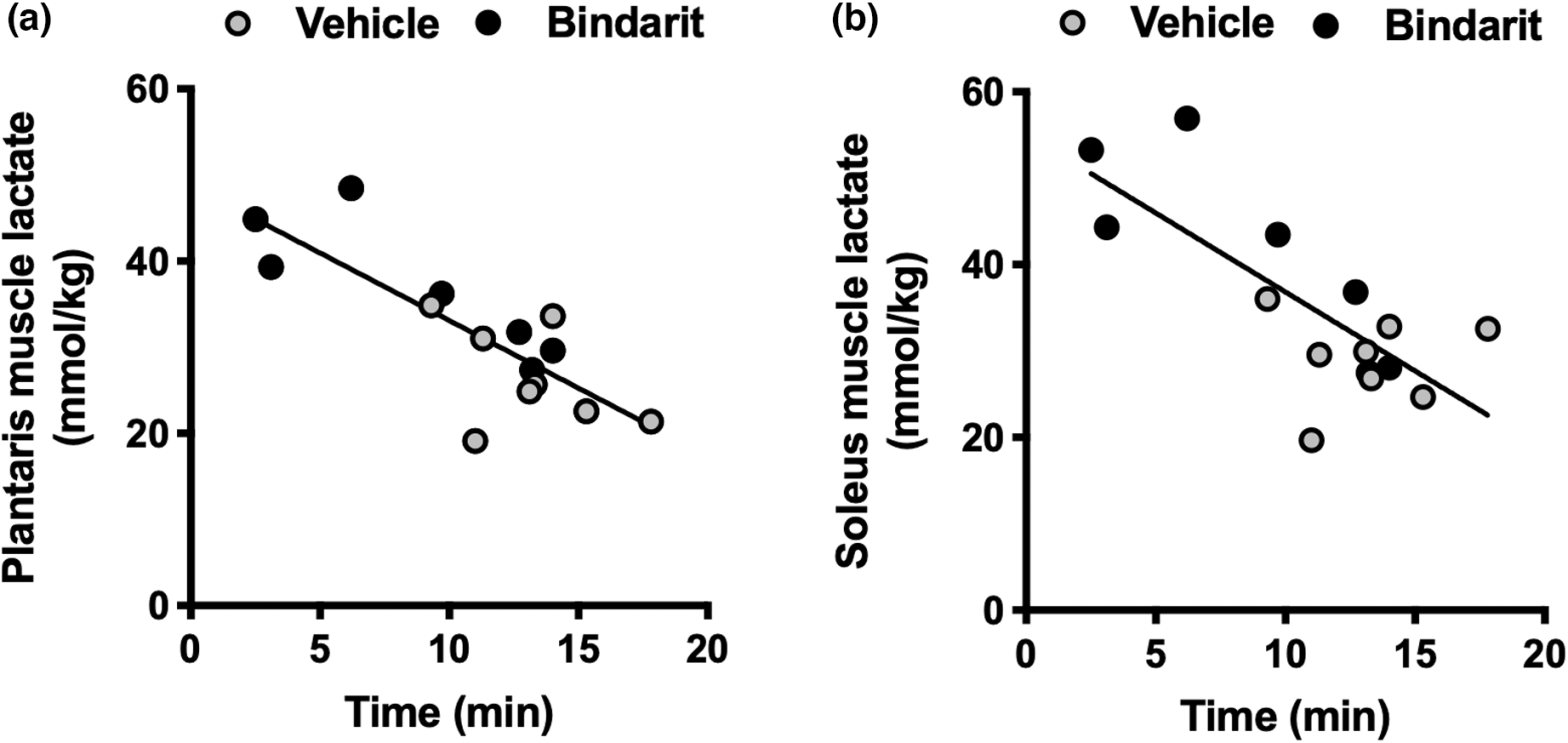
Correlations between the running duration in treadmill running test at a speed of 40 m/min and (a) plantaris (*r* = −0.80, *p* < 0.01) and (b) soleus (*r* = −0.75, *p* < 0.01) muscle lactate concentration after exercise among all samples in Experiment 2.

Considering that MCT4 is a low‐affinity lactate transporter, we performed treadmill running at 40 m/min. There was a trend of difference in the running survival curves (*p* = 0.10, Figure 4a). The average running duration was significantly reduced in mice post‐bindarit administration (Figure 4e). The blood lactate (Figure 4b) and glucose (Figure 4f) levels after exercise did not differ between the groups. Muscle lactate concentrations after exercise were significantly higher in bindarit‐administered mice than that in vehicle‐administered mice, both in the plantaris (Figure 4c) and soleus (Figure 4g). Similar glycogen depletion was observed in the plantaris (Figure 4d) and soleus (Figure 4h). There were significant negative correlations between the exercise duration and muscle lactate concentration in the plantaris (Figure 5a) and soleus (Figure 5b).

## 4 DISCUSSION

Lactate was once thought to be associated with fatigue during exercise, but is now seen as an important energy substrate. It not only serves as the link between glycolytic and oxidative metabolism within a cell but is also shuttled between cells and tissues (Brooks, 2018). Although the lactate shuttle theory (Brooks, 1986) is now widely recognized, the role of lactate during exercise is not fully understood. In the current study, we examined whether selective inhibitors of MCT1 and MCT4 alter the exercise capacity and lactate metabolism. Our findings of reduced exercise duration after MCT inhibition provide confirmation that lactate transport plays a pivotal role during exercise.

MCT1 and MCT4 are predicted to have 12 transmembrane domains in common, but the amino acid sequence alignments showed identities of only 42% in mice and 44% in humans (BLAST on NCBI), indicating that these transporters have different properties. Among a variety of MCT inhibitors described in the literature, 4‐CIN is the most frequently used and has been shown to inhibit lactate transport in skeletal muscle (Mcdermott & Bonen, 1994; Roth & Brooks, 1993; Wibrand & Juel, 1994). This classic inhibitor is known to reversibly inhibit MCT1 with more than 5‐fold selectivity compared with MCT4 (Payen et al., 2020). In contrast, bindarit, a recently identified MCT4 inhibitor, has been shown at least 15‐fold selectivity compared with MCT1 (Futagi et al., 2018). In this study, we employed these inhibitors to assess the role of MCT1 and MCT4 during exercise.

To confirm the inhibition of MCT1, we first showed that the blood lactate concentration increased after 4‐CIN administration, indicating that lactate uptake into tissues is impaired. Since both human (Mazzeo et al., 1986) and mouse (Kitaoka et al., 2016) studies have shown that blood lactate concentration declines rapidly after lactate injection under normal conditions, we evaluated the time‐dependent changes in blood lactate concentration during LTT in 4‐CIN‐administered mice. The area under the curve for blood lactate concentration also supported the effectiveness of this inhibitor. Using this MCT1 inhibitor, we demonstrated a reduction in the treadmill running duration at 20 m/min. The increased blood lactate concentration in 4‐CIN‐administered mice was in agreement with our observations in LTT. We have previously shown that the blood lactate concentration during LTT was lower in exercise‐trained mice than that in the untrained mice (Takahashi et al., 2019). Lactate is an important energy substrate during exercise, and lactate transport capacity is reported to increase in response to chronic muscle contraction (McCullagh et al., 1996), whereas it decreases in denervated muscles (Mccullagh & Bonen, 1995). Taken together, our results suggest that lactate uptake into tissues, we assume mainly skeletal muscle, is important for sustaining endurance exercise. However, it should be noted that muscle lactate concentration was higher in 4‐CIN‐administered mice compared to that in exercise duration‐matched control mice. Considering that MCT1 is localized not only in plasma membranes but also in mitochondrial membranes (Butz et al., 2004), we speculate that 4‐CIN administration may affect lactate/pyruvate transport into the mitochondria. This decrease in lactate/pyruvate oxidation may explain the marked decline in exercise performance induced by 4‐CIN.

Unlike MCT1 (Km: 3.5 mmol/L), MCT4 has low lactate affinity (Km: 28 mmol/L), MCT4 is thought to be suitable for outward lactate transport from glycolytic cells (Halestrap, 2013). We have shown that decreased training intensity reduces exercise performance and MCT4, despite maintaining oxidative capacity and MCT1 in the skeletal muscle of Thoroughbred horses (Kitaoka et al., 2011). These observations led us to hypothesize that MCT4 is important for high‐intensity exercise performance. In this regard, a treadmill running test at 40 m/min showed a reduction in running duration in MCT4 inhibitor bindarit‐treated mice. Our results indicated that lactate extrusion from the muscle is crucial for maximal exercise. This is consistent with our previous report showing a correlation between MCT4 protein levels in skeletal muscle and exercise duration in an incremental all‐out test in Thoroughbreds (Kitaoka et al., 2010). To ascertain the importance of MCT4, we measured the increase in lactate concentration in the muscle immediately after exercise. Intriguingly, exercise duration was negatively correlated with muscle lactate concentrations, further supporting the importance of lactate extrusion from the muscles. We surmise that MCT4 enables skeletal muscles to maintain high rates of glycolytic ATP production during exercise.

Although a number of studies have shown that exercise training increases MCT1 and MCT4 expression in skeletal muscle (Thomas et al., 2012), data describing the effects of inhibition of these transporters on exercise capacity are scarce. In this study, we found a significant reduction in treadmill running duration after MCTs inhibition which indicates that lactate transport is essential for sustaining exercise. However, the present work is not without limitations. Although we aimed to focus on skeletal muscle, our experimental model cannot deny that the inhibitors may affect other tissues, considering that MCT1 and MCT4 are widely expressed throughout the body (Bonen et al., 2006). Furthermore, the effectiveness of pharmacological inhibition of MCTs may be not identical in various tissues. It is noteworthy that multiple tissues contribute to systemic lactate turnover during exercise (Van Hall, 2010). Another limitation of this study is the specificity of inhibitors. Although 4‐CIN is considered as a classic inhibitor of MCT1 because of its major role in its discovery, it is also described to inhibit other MCTs and mitochondria pyruvate carrier (MPC), depending on the concentration (Halestrap, 2013). Moreover, bindarit is reported to be a highly selective and non‐competitive inhibitor of MCT4 (Futagi et al., 2018), but it has also been known as an anti‐inflammatory agent that down‐regulates NF‐κB pathway (Mora et al., 2012). Future studies are needed to use other inhibitors, such as AZD3965 (Beloueche‐Babari et al., 2020). Recently, global MCT4 knockout mice have been shown to exhibit an exercise intolerant phenotype (Bisetto et al., 2019), whereas MCT1 knockout mice are embryonically lethal (Lengacher et al., 2013). In future studies, it is necessary to investigate skeletal muscle‐specific knockdown or knockout models to assess the contribution of MCTs more specifically.

## CONFLICT OF INTEREST

The authors have no conflicts to declare.
