# Correction to Inhibition of monocarboxylate transporters (MCT) 1 and 4 reduces exercise capacity in mice

**DOI:** 10.14814/phy2.16031

**Published:** 2024-05-03

Kitaoka Y , Takahashi K , Hatta H . Inhibition of monocarboxylate transporters (MCT) 1 and 4 reduces exercise capacity in mice. Physiol Rep. 2022;10:e15457.36065874 10.14814/phy2.15457PMC9446403

In the above article, the dose of α‐cyano‐4‐hydroxycinnamate (4‐CIN) was incorrectly shown as per gram of body weight instead of per kilogram of body weight throughout the manuscript. The correct unit is mg/kg. The conclusions of the study are not affected by this correction.

We apologize for this error.

